# *Klebsiella* Phage vB_KleM-RaK2 — A Giant Singleton Virus of the Family *Myoviridae*

**DOI:** 10.1371/journal.pone.0060717

**Published:** 2013-04-09

**Authors:** Eugenijus Šimoliūnas, Laura Kaliniene, Lidija Truncaitė, Aurelija Zajančkauskaitė, Juozas Staniulis, Algirdas Kaupinis, Marija Ger, Mindaugas Valius, Rolandas Meškys

**Affiliations:** 1 Department of Molecular Microbiology and Biotechnology, Institute of Biochemistry, Vilnius University, Vilnius, Lithuania; 2 Laboratory of Plant Viruses, Institute of Botany, Nature Research Centre, Vilnius, Lithuania; 3 Proteomics Centre, Institute of Biochemistry, Vilnius University, Vilnius, Lithuania; Centro Nacional de Biotecnologia - CSIC, Spain

## Abstract

At 346 kbp in size, the genome of a jumbo bacteriophage vB_KleM-RaK2 (RaK2) is the largest *Klebsiella* infecting myovirus genome sequenced to date. In total, 272 out of 534 RaK2 ORFs lack detectable database homologues. Based on the similarity to biologically defined proteins and/or MS/MS analysis, 117 of RaK2 ORFs were given a functional annotation, including 28 RaK2 ORFs coding for structural proteins that have no reliable homologues to annotated structural proteins in other organisms. The electron micrographs revealed elaborate spike-like structures on the tail fibers of Rak2, suggesting that this phage is an atypical myovirus. While head and tail proteins of RaK2 are mostly myoviridae-related, the bioinformatics analysis indicate that tail fibers/spikes of this phage are formed from podovirus-like peptides predominantly. Overall, these results provide evidence that bacteriophage RaK2 differs profoundly from previously studied viruses of the *Myoviridae* family.

## Introduction

Bacteriophages (phages) represent the largest reservoir of unexplored genetic information in the biosphere. With biochemical or genetic data being available for a few prototypical phages only, there is a bewildering array of different genomes to characterize [1], [2], [3], [4]. Tailed phages, representing the most diversified of all virus groups, constitute the order Caudovirales with three families, characterised by contractile, long and noncontractile, or short tails and named *Myoviridae*, *Siphoviridae* and *Podoviridae,* respectively [4]. The myoviruses generally have larger genomes than bacteriophages representing other phage families [5]. Moreover, there is a handful of myoviruses with sequenced genomes in the 200 to 500 kbp range. These phages are usually referred to as “giant phages” or “jumbo phages” [6], and include such myoviruses as: *Pseudomonas aeruginosa* phages phiPA3 (309 kbp, [7]) and, phiKZ (280, [8]), *Ralstonia* phage phiRSL1 (231 kbp, [9]), *Yersinia enterocolitica* phage R1-37 (270 kbp, [10]). The largest myovirus sequenced to date is *Bacillus* phage G (498 kbp, #JN638751) followed by *Cronobacter* phage vB_CsaM_GAP32 (359 kbp, #JN882285), yet the analysis of these sequences has not been published so far. It is worth mentioning that the vast majority of sequenced giant bacteriophages have no close relatives [6]. Therefore, the evolutionary relationships between these phages and other well-studied myoviruses are difficult to determine. Moreover, in the case of the jumbo phages, the genome anotation using conventional methods (such as comparative genomics or other similar approaches that have proven useful in studying smaller phages) is seriously hampered by a large number of orphan genes. Hence, compared to their smaller brethren, the giant bacteriophages have been less thoroughly researched [6].

Myoviruses infect a broad range of bacterial hosts, including bacteria that prevail in aquatic environments (e.g. cyanobacteria) as well as these that are associated with various human diseases (e.g. pathogenic strains of *Escherichia coli*, *Pseudomonas*, *Shigella*, *Klebsiella* etc.). *Klebsiella* are ubiquitously present worldwide. In addition to being the primary cause of various respiratory tract infections, they are frequently associated with the infections of the alimentary, urinary and genital tracts [11]. Moreover, due to the widespread use of antibiotics, multidrug-resistant *Klebsiella* strains emerge rapidly [12], [13], [14]. In the past few years, several lytic bacteriophages have been reported to be useful in controlling and detection of this bacterial species, yet studies concerning newly isolated *Klebsiella* phages have almost exclusively focused on biological characteristics or biodistribution and therapeutic efficacy in animal models [15], [16], [17]. Moreover, of all the tailed phage genome sequences present in the NCBI database to date, only one is of *Klebsiella*-specific lytic myovirus, bacteriophage KP15 (174 kbp, #GU295964). Therefore, in the case of this particular group of bacterial viruses, only limited genomic and proteomic data are available, and the molecular mechanisms underlying phage-host interaction are rather obscure [18], [19], [20].

We present here a novel bacteriophage vb_KleM-Rak2 (Rak2), isolated using a multidrug-resistant *Klebsiella* sp. veterinary isolate KV-3. Unique morphological and physiological characteristics as well as genomic and proteomic features have been described. With a genome size of 345,809 bp, Rak2 is the third largest myovirus and the largest *Klebsiella* phage sequenced to date. Moreover, limited homology on the DNA and protein levels indicate that RaK2 has no close phage relatives and therefore is a singleton virus of the family *Myoviridae*.

## Materials and Methods

### Phages and Bacterial Strains

Bacteriophage RaK2 was originally isolated from sewage-polluted pond in Lithuania using *Klebsiella* sp. veterinary isolate KV-3 as the host for phage propagation and phage growth experiments [21]. Bacterial strains used in this study for host range determination are listed in Table S1 [22], [23], [24], [25].

MCIC [26] and SCAI [27] selective media were used for isolation of *Klebsiella* sp. related bacterial strains. For phage experiments, bacteria were cultivated in Luria-Bertani broth (LB) or LB agar. Bacterial growth was monitored turbidimetrically by reading OD_600_. An OD_600_ of 0.4 corresponded to 1.4×10^7^ KV-3 cells/ml.

To identify the isolates, PCR-amplified 16S rRNA gene sequence analysis was performed. Universal primers woo1 and woo2 [28] as well as the sequence specific primers Klebs16S_F1 5′–GTGACATGGATTCTTAACG–3′ and Klebs16S_R1 5′-GTGTGTGGTTTCAATTTTC–3′) were used both for PCR amplification and subsequent sequencing of the target 16S RNA gene.

### Phage Techniques

For host range determination, a double agar overlay plaque assay method [29] was employed. Phage isolation, plating and titering were carried out as described previously [30]. Further purification using a CsCl step gradient was performed [31] with few modifications. The phage suspension was deposited on the top of CsCl step gradient (densities: 1.1 g/ml, 0.9 g/ml, 0.7 g/ml, 0.5 g/ml) and centrifuged in a Spinco SW39 rotor for 3 h at 24,000 rpm, 11°C. The resulting phage band with the highest opalescence was collected with a syringe and dialyzed against three changes of SM buffer (100 mM NaCl, 8 mM MgSO_4_, 50mM Tris-HCl, pH 7.5) at 4°C. The adsorption tests and one-step growth experiments were carried out as described by Khusainov et al. [32] and Carlson and Miller [33], respectively. Meanwhile determination of the efficiency of plating (e.o.p.) was performed as described previously [34]. High-titer phage stocks were diluted and plated in duplicate. Plates incubated at 18, 22, 24, 26, 28, 30, 32, 34 and 36°C were read after 24–48 hours of incubation. The temperature at which the largest number of plaques were formed was taken as the standard for the e.o.p. calculation.

### Transmission Electron Microscopy (TEM)

CsCl density gradient-purified phage particles were diluted to approximately 10^11^ PFU/ml with distilled water, 5 µl of the sample were directly applied on the carbon-coated nitrocellulose grids, excess liquid was drained with filter paper before staining with two successive drops of 2% uranyl acetate (pH 4.5), dried and examined in Morgagni™ 268(D) transmission electron microscope (FEI, Oregon, USA). Rak2 virions were measured (30 virions in total) after calibration with catalase crystals and/or T4 phage particles.

### DNA Isolation

Aliquots of phage suspension (10^11^–10^12^ PFU/ml) were subjected to phenol/chloroform extraction and ethanol precipitation as described by Carlson and Miller [33]. Isolated phage DNA was subsequently used in restriction analysis, for PCR or was subjected to genome sequencing.

### Genome Sequencing and Analysis

The complete genome sequence of RaK2 was determined using HiSeq 2000 DNA sequencing technology and primer walking. Open reading frames (ORFs) were predicted with Glimmer v2.02 (http://nbc11.biologie.uni-kl.de). Analysis of the genome sequence was performed using the Fasta-Protein, Fasta-Nucleotide, Fasta-Genome, BLAST2, PSI-Search, Transeq and ClustalW2 programs. (http://www.ebi.ac.uk), HHsearch1.6 (http://toolkit.tuebingen.mpg.de/hhpred), COMA (http://bioinformatics.ibt.lt:8085/coma), Sequence editor (http://www.fr33.net/seqedit.php) and Geneious v5.5.6. (Société Geneious, Colombes, France). tRNAscan-SE 1.21 (http://lowelab.ucsc.edu/tRNAscan-SE/) was used to search for tRNAs. Phylogenetic and molecular evolutionary analyses were conducted using MEGA version 5 [35]. Genome-wide search for regulatory sequences was performed using extractUpStreamDNA (http://lfz.corefacility.ca/extractUpStreamDNA/), MEME analysis [36] at http://meme.sdsc.edu/meme/cgibin/meme.cgi as well as PePPER (http://pepper.molgenrug.nl/index.php/pepper-tools) and SoftBerry (http://linux1.softberry.com/berry.phtml).

### DNA Methylation Analysis

Investigation of RaK2 DNA methylation was performed using bisulfite conversion and subsequent sequencing. Bisulfite modification of genomic DNA was performed by using the EZ DNA Methylation-Gold™ Kit (Zymo Research, Irvine, USA) according to manufacturers protocols.

### Analysis of Structural Proteins

CsCl-purified phage particles were separated on 12% SDS-PAGE. Protein bands were visualized by staining the gel with PageBlue™ Protein Staining Solution (Thermo Fisher Scientific, Vilnius, Lithuania) and excised from the gel using a razor blade. Each sample was purified as described in [37] with minor modifications. In short, gel slices were destained with 50 mM ammonium bicarbonate in 50% acetonitrile (ACN), vacuum-dried, rehydrated in 100 µl of trypsin (20 µg ml^–1^) (EMP Biotech, Berlin, Germany) containing 25 mM ammonium bicarbonate in 9% ACN, covered with additional 75 µl of 25 mM ammonium bicarbonate in 9% ACN and incubated at 37°C overnight. The peptides were extracted from the gel using 100 µl of 5% trifluoracetic acid in 50% ACN for 30 min. This extraction procedure was repeated twice. The peptides from the two extractions were combined, concentrated by vacuum drying, resuspended in 2% ACN with 0.1% trifluoracetic acid to 40 µl. and subjected to proteomic analysis.

### Liquid Chromatography of Tryptic Peptides

Reversed-phase (RP) nano-liquid chromatography directly coupled with mass spectrometry (LC-MS/MS) was performed using an Ultimate 3000 nano-flow LC system (Thermo Scientific, Sunnyvale, USA) connected to QTRAP 4000 (AB Sciex, Framingham, USA). Peptides were loaded on a RP trap column PepSwift PS-DVB, 200 µm (Thermo Scientific, Sunnyvale, USA) with a flow-rate of 20 µl/min (loading buffer: 2% ACN and 0.1% trifluoracetic acid) and subsequently separated by high performance liquid chromatography (HPLC) on an Acclaim PepMap 100 C18 (Thermo Scientific, Sunnyvale, USA) analytical column (75 µm×15 cm, 3 µm bead size) in 50 min linear gradient (A: 0.05% trifluoracetic acid, B: 80% ACN and 0.04% trifluoracetic acid) at a flow rate of 300 nl per min.

### Mass Spectrometry

Tryptic-digest from each gel slice was analyzed by 4000 QTRAP (AB Sciex, Framingham, USA) mass spectrometry in linear ion trap mode using information dependent acquisition (IDA) and dynamic exclusion protocol. The acquisition method consisted of an IDA scan cycle including the enhanced mass scan (EMS) as the survey scan, enhanced resolution scan (ER) to confirm charge state and six dependent enhanced product ion (EPI) scans (MS/MS). With the threshold of the ion intensity at 100000 counts per second (cps), the IDA criteria were set to allow the most abundant ions in the EMS scan to trigger EPI scans. Survey MS scan was set to mass range from 400 m/z to 1400 m/z. Dynamic ion exclusion was set to exclude precursor ions after their two occurrences during 60 s interval. Peak lists were generated using Analyst software 1.4.2 (AB Sciex, Framingham, USA) and Mascot search script 1.6b9 and searched with Mascot v2.2.07 (Matrix Science, Boston, USA) against the the in-house protein database derived from vB_KleM-RaK2 genome sequences. Each of the peak lists were searched using Mascot algorithm (p-value of 0.05) for full tryptic peptides using a precursor ion tolerance window set at ±0.5 Da, variable methionine oxidation and NQ deamidation and maximally one missed cleavage were allowed. The maximum fragment ion tolerance (MS/MS) was ±0.35 Da.

### Nucleotide Sequence Accession Numbers

All new data has been deposited in GenBank. The complete genome sequence of *Klebsiella* bacteriophage RaK2 was deposited in the EMBL nucleotide sequence database under accession number JQ513383. The accesion numbers for the 16S RNA sequences of bacteria strains used for host range determination are as follows: *Buttiauxella* sp. S1-1 (JX406856), *Enterobacter* sp. VT1-1 (JX406857), *Klebsiella* sp. KV-1 (JX406858), *Klebsiella* sp. KV-3 (JX406859), *Pseudomonas* sp. PV1-1 (JX406860), *Pseudomonas* sp. RA1-1 (JX406861), *Pseudomonas* sp. RA1-3 (JX406862), *Pseudomonas* sp. RA1-11 (JX406863).

## Results

### Virion Morphology

**Figure 1:**
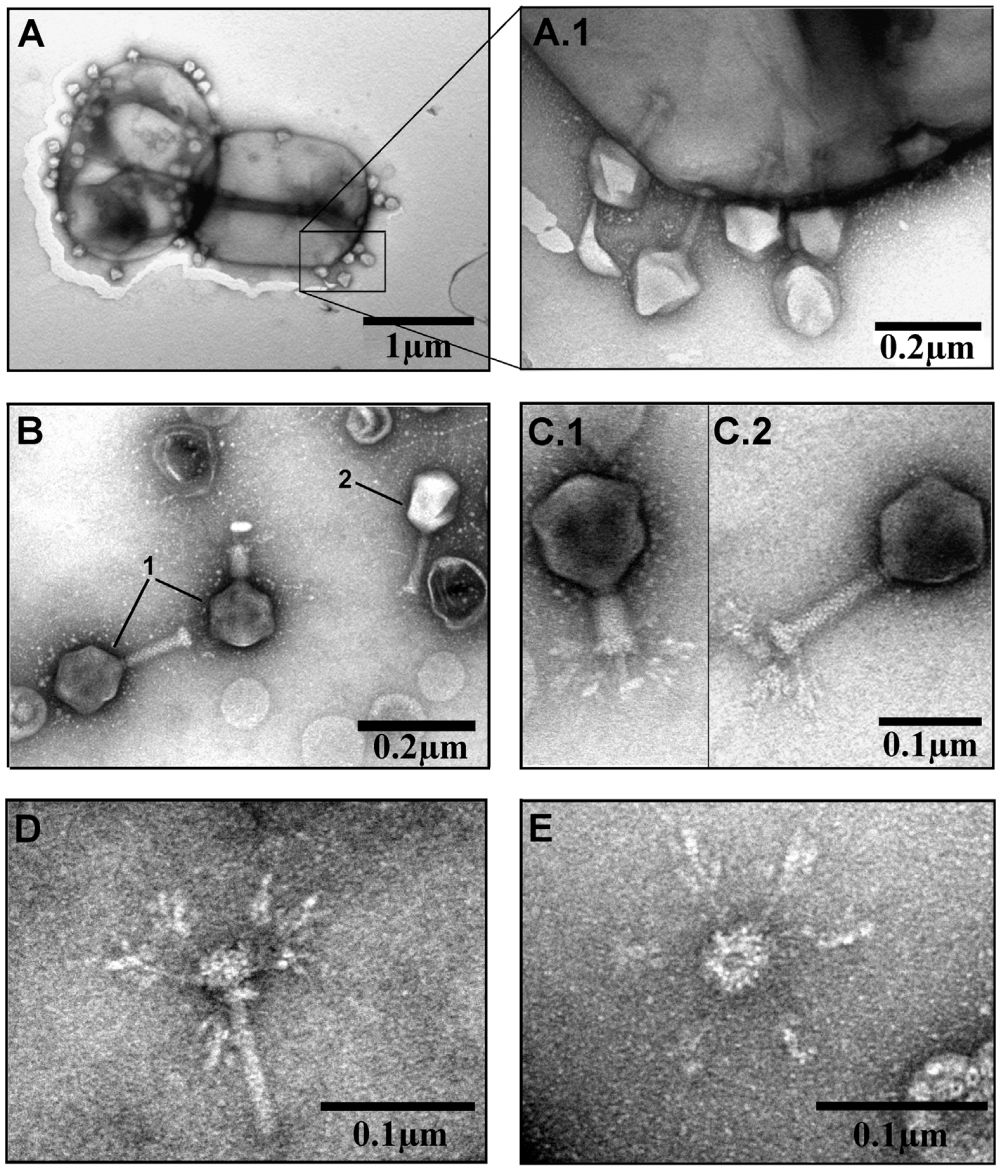
Electron micrographs of *Klebsiella* phage RaK2. (A and A.1) Phage RaK2 particles adsorbed to the surface of *Klebsiella* sp. KV-3 cells. (B) Purified phage RaK2 particles (1) and one particle of phage T4 (2). (C) RaK2 particle with contracted (C.1) and extended (C.2) tail. (D) Inner tail tube with baseplate and baseplate-associated ramified tail fiber structures. (E) Baseplate with six long tail fibers.

Transmission electron microscopy revealed that phage RaK2 (Fig. 1) belongs to the family *Myoviridae* (A1 morphotype), and is characterized by an isometric head of 123 nm in diameter and a contractile tail (128 x 21.5 nm). The phage has a clearly defined neck (12.3 nm), baseplate (35 nm), collar (18.5 nm) and six baseplate-associated ramified long tail fibers. Due to the morphological intricacy, the length of tail fibers of RaK2 could not be measured. As may be seen in Fig. 1, the long tail fibers of this virus are generously studded with spike-like structers, making it impossible to accurately determine the actual length of tail fiber of RaK2.

It is worth mentioning that superficially, tail fibers of Rak2 resemble those found in viunalikeviruses (ViI-like phages), a genus that has been proposed only recently [38]. In contrast to viunalikeviruses, however, the adsorption organelle of Rak2 does not seem to undergo any conformational changes, and no “quiescent tails” [38] have been observed for this phage.

### The Host Range of RaK2

In total, 40 bacterial strains (Table S1) were used to explore the host range of RaK2. With the exception of *Klebsiella* sp. veterinary isolate KV-3, all of 4 *Acinetobacter* sp., 5 *Arthrobacter* sp., 1 *Buttiauxella* sp., 1 *Citrobacter* sp., 2 *Enterobacter* sp., 2 *Erwinia* sp., 11 *Escherichia* sp., 6 *Pseudomonas* sp., 2 *Rhodococcus* sp., 1 *Salmonella* sp. and 4 *Klebsiella* sp. strains were found to be resistant to RaK2. *Klebsiella* sp. veterinary isolate KV-3 was isolated from pig farm sewage environmental samples. This isolate was found to be susceptible to kanamycin (10 µg/ml), gentamicin (8 µg/ml) and neomycin (10 µg/ml) but resistant to ampicillin (60 µg/ml), streptomycin (50 µg/ml), tetracycline (50 µg/ml) and moderately resistant to chloramphenicol (up to 25 µg/ml). Although a number of strains used in this study is not large, the diversity of bacteria tested allow presuming that the host range of Rak2 is limited to *Klebsiella* only.

### Phage Growth Characteristics

In order to determine the optimal conditions for the one-step growth experiment, both the e.o.p. and adsorption tests were performed. The e.o.p. of RaK2 was examined in the temperature range of 18–37°C (data not shown). This test revealed that the phage has an optimum temperature for plating around 30°C. Interestingly, at temperatures below 22°C or above 34°C, small and barely visible plaques of Rak2 have been observed (data not shown). Adsorption tests showed that a high percentage (93%) of the RaK2 particles adsorb to *Klebsiella* sp. veterinary isolate KV-3 cells after 10 min of incubation.

**Figure 2:**
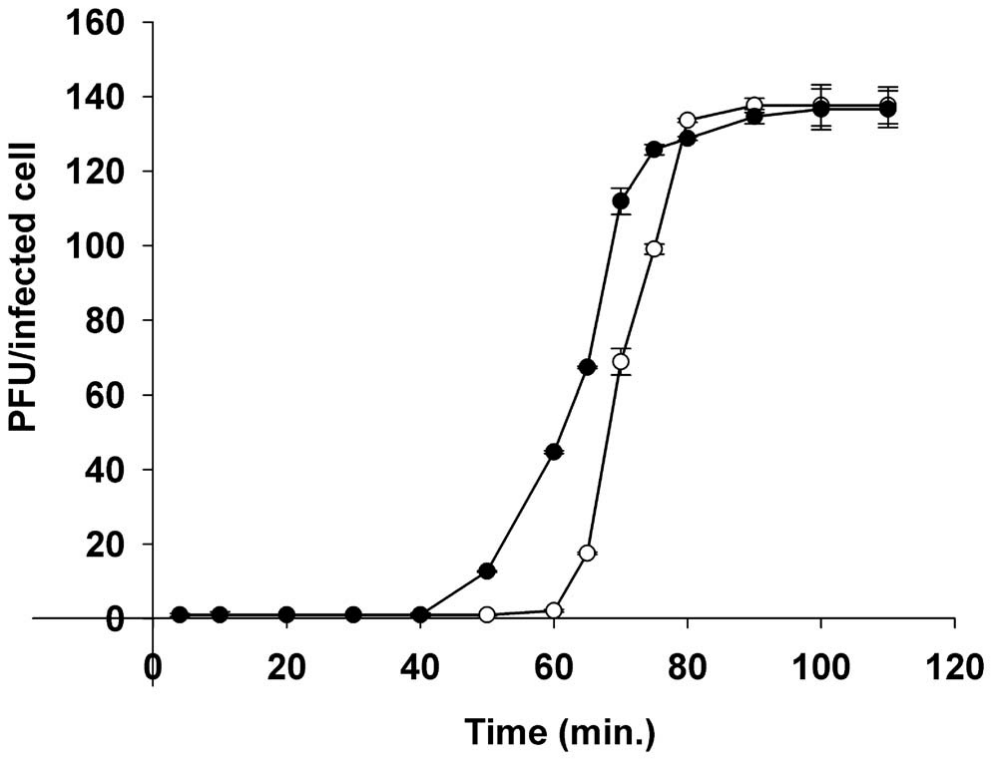
Single-step growth curves of bacteriophage RaK2 at 30°C. Shown are PFU per infected *Klebsiella* sp. KV-3 cell in chloroform-treated cultures (filled circle) and untreated cultures (circle) at different time points. Each point represents the mean of three individual experiments.

Subsequently, the infection cycle of RaK2 was studied by performing single-step growth experiments at 30°C as described in “Materials and methods”. The curves obtained indicate (Fig. 2) that the latent period of RaK2 is 60 min, the eclipse period ∼40 min and the phage generates an average burst of ∼140 virions per infected cell.

**Figure 3:**
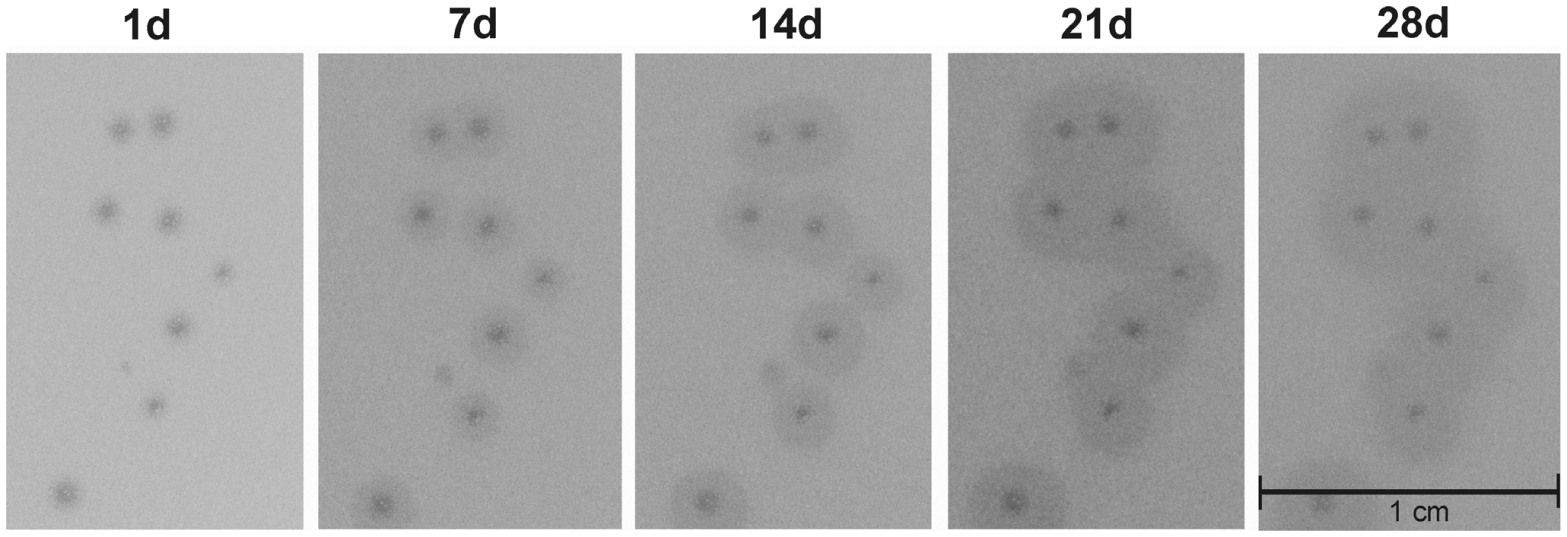
Growth of RaK2 plaques at 30°C. Numbers above indicate days of incubation.

In the course of the experiments mentioned above, we noticed that RaK2 produces small clear plaques surrounded by constantly, albeit slowly, growing opaque halo zones (Fig. 3). After one day (24 h) of incubation at 30°C, bacteriophage RaK2 forms plaques of 0.75±0.25 mm in diameter, after 7 days 1.75±0.25 mm, after 14 days 2.25±0.75 mm, after 21 days 3.25±1.25 mm and the plaques reach 3.30±0.75 mm in diameter within a period of 28 days.

### Genome Analysis of RaK2

The genome sequence analysis revealed that RaK2 has a total of 534 probable protein-encoding genes [21]. However, based on the similarity to biologically defined proteins, only 79 of RaK2 ORFs were given a functional annotation (Table S2) and are described in greater detail in the following sections.

### DNA Replication, Recombination and Repair Proteins

RaK2 encodes PolB family (PolB-type) DNA polymerase (gp100), which is closely related to gp43 of vibriophage KVP40 (a distant relative of phage T4) belonging to the subfamily *Tevenvirinae*. The replisome complex of Tevenviruses is highly conserved and consists of DNA polymerase (gp43), sliding clamp loader (gp44 and gp62), sliding clamp (gp45), DNA helicase (gp41), DNA primase (gp61), and single-strand DNA binding (gp32) proteins [39], [40]. With the exception of gp45 and gp62, all of the proteins listed above have distant homologues in Rak2. Additionally, RNase H (gp108) and DNA ligase (gp486), required to join Okazaki fragments, are also encoded by RaK2. In contrast, no apparent homologue of primase-helicase loader was detectable in the genome of the phage analyzed.

Based on the amino acid sequence similarity, five gene products of Rak2 have been assigned as proteins required for DNA recombination and repair, including both recombination endonuclease subunits (gp084 and gp083) as well as RecA-like protein (gp114), helicase/ATP-ase (gp118) and a homologue of vb_EcoM-VR7 pyrimidine dimer N-glycosidase (endonuclease V; gp291) that is used for the repair of UV irradiation-induced DNA damage [41]. It is worth noting that Rak2 also encodes a distant homologue of endoVII packaging and recombination endonuclease of *Salmonella* phage PVP-SE1 [42]. However, no characterized recombination mediator proteins (UvsY) have detectable homologues in the genome of Rak2.

### Nucleotide Metabolism and DNA Modification Enzymes

In total, nine characterized enzymes for deoxynucleoside triphosphate conversion and pyrimidine synthesis have distant homologues in RaK2, including thymidine kinase (gp021), dNTP kinase (gp040), dTMP synthase (gp059), dCMP deaminase (gp495), dihydrofolate reductase (gp148) as well as aerobic and anaerobic ribonucleoside diphosphate reductase subunits (corresponding to NrdA/B (gp522/gp141 and NrdD/G (gp520/gp523) respectively). However, in the absence of the detectable homologs to enzymes such as dUTPase or dCTPase/dUTPase, thioredoxin and anaerobic ribonucleoside diphosphate reductase subunit H (NrdH), the metabolic pathway used by RaK2 in the conversion of ribonucleotides to deoxyribonucleotides (or/and dCMP to dTTP) remains unclear.

In order to protect the genomic DNA from restriction endonucleases, phages employ various strategies including adenine and cytosine methylation as well as hydroxymethylation of cytosine (HMC) and subsequent glucosylation of HMC derivatives [43], [44]. The presence of aparent homologs to the characterized dCMP hydroxymethylases and glucosyltransferases (e.g. a-gt, b-gt) has not been confirmed in RaK2, suggesting that this phage neither utilizes HMC nor employs DNA glucosylation.

Nevertheless, two putative DNA-methyltransferases (gp194 and gp447) encoded by RaK2 indicate that this phage may have a different strategy for DNA modification. This hypothesis is supported by the results of restriction analysis (data not shown), revealing that the DNA of Rak2 can only be digested by the restriction enzymes which are known to recognize Dam, Dcm, CpG and EcoKI methylated DNA. In addition, the analysis of bisulfite-treated DNA (data not shown) revealed that the DNA of RaK2 contains methylated C^m^pG at nucleotide position 5′-.

### tRNAs and tRNA-related Enzymes

The RaK2 genome has a substantially lower G+C content than its host (32% v. 57%), therefore it is possible that in order to ensure the effective rate of translation, this phage needs to direct the synthesis of its own tRNAs. RaK2 encodes five apparently functional tRNA genes (tRNA^Asn^, tRNA^Arg^, tRNA^Thr^, tRNA^Ser^ and tRNA^Ser2^) and two pseudo-tRNA genes (tRNA^His^ and tRNA^Val^) organized into two adjacent ∼4 kb and ∼1 kb clusters, respectively. However, only three out of five Rak2 tRNAs recognize codons with A or U in the third position.

RaK2 encodes a putative CCA-adding enzyme (gp031), which could function in the maturation of its own tRNAs, as well as two RNA repair proteins, namely polynucleotide kinase (gp470) and RNA ligase (gp464). Although a number of tRNAs encoded by Rak2 is not large, under certain conditions a tRNA repair pathway would be perhaps beneficial to this phage.

It is worth mentioning that RaK2 also encodes a putative peptidyl-tRNA hydrolase type 2 (ORF188 (Pth2)). Interestingly, there are two structurally different enzymes that have been reported to encode such activity, Pth present in bacteria and eucaryotes, and Pth2 present in archaea and eucaryotes [45].

### Lysis, Host or Phage Interactions

The genome of RaK2 encodes three potential lysozymes, namely ORF029, ORF073 and ORF078. Although the deduced amino acid sequences of these proteins vary in length and composition, a comparative sequence analysis revealed that the lysozyme domain of all the enzymes listed above share a high percent similarity (54% ORF029 and ORF073, 50% ORF029 and ORF078 and 66% ORF073 and ORF078). In addition, gp029 and gp073 of Rak2 are similar to lysozymes from *Pseudomonas* phage tf and *Pseudomonas* phage LUZ24, respectively. Meanwhile the lysozyme domain of Rak2 gp078 shares 28% identity with tail lysozyme from T4-like virus phage KP15.

Rak2 ORF078 encodes one of the largest base-plate associated lysozymes (886 aa) identified to date and contains a very unusual domain composition. Tail–associated lysozyme of bacteriophage T4 (575 aa), the product of gene 5 (gp 5), is an essential structural component of the hub of the phage. This protein consists of three domains: an N-terminal oligosaccharide binding-fold domain, the middle lysozyme domain, and the C-terminal triple β-helix domain [46]. Gp078 of Rak2 is much larger in size (886 aa) and consists of the β-helix domain flanked by two regions that have no apparent database matches and the C-terminal lysozyme domain.

Bacteriophage Rak2 also harbours two proteins (gp015 and gp496) which may be involved in phage-host interaction. The product of ORF496 shares 38% amino acid sequence identity with gp008 (conserved structural protein containing bacterial Ig-like and invasin/intimin cell-adhesion domain) of *Erwinia* phage vB_EamM-M7 [47]. Meanwhile the protein encoded by ORF015 exhibits a moderate sequence identity (34%) to RI.-1 of T4-related *Aeromonas* phage Aeh1. It was suggested that in T4, two accessorry proteins RI.-1 and RI.1, together with a protein RIII, participate in the regulation of lysis inhibition [48]. However, since Rak2 not only lacks the apparent homologs of RI.1 and RIII, but the T holin and RI antiholin are absent as well, the ability of this phage to elicit the phenomenon of lysis inhibition seems rather questionable.

### Structural Proteins

To identify structural genes of Rak2, MS/MS-based proteomics and bioinformatics approach was undertaken. Comparative analysis of the genome sequence of Rak2 allowed the annotation of 21 structural genes, including those coding for head (ORF094, ORF100), neck (ORF038, ORF089, ORF530), tail (ORF078, ORF071w, ORF067w, ORF041, ORF087, ORF088, ORF089, ORF139), tail fiber/tail spike (ORF098, ORF527, ORF528, ORF531, ORF532, ORF533) and other morphogenesis-associated proteins. In agreement with morphological data, the majority of Rak2 structural proteins listed above exhibit detectable sequence similarity to proteins encoded by other myoviruses, especially cyanobacterial myoviruses. Most of the tail fiber/tail spike proteins of Rak2, however, show sequence homology only with proteins of phages of the family *Podoviridae*.

Reversed-phase nano-liquid chromatography directly coupled with LC-MS/MS analysis of the structural Rak2 proteins separated by SDS PAGE led to the experimental identification of 54 virion proteins (Table S3), including 19 that were predicted by bioinformatics approaches and 28 unique proteins, which either have no detectable homology to any entries in the public databases or are similar to hypothetical proteins from vB_CsaM_GAP32 only (Table S2). In addition, the MS analysis showed two unexpected proteins, including prohead core scaffolding protein/protease (gp092) and a single stranded DNA binding protein (gp113) with a low, yet significant peptide coverage (>9.6%). In the case of large viruses, so-called “unexpected” proteins are often being identified [49], [50]. Although the identification of such proteins is always puzzling, one must bear in mind that the extensive molecular analysis so far has been performed in the case of the few prototype bacterial viruses only, providing very limited insight into the biology of the broader phage population.

**Figure 4:**
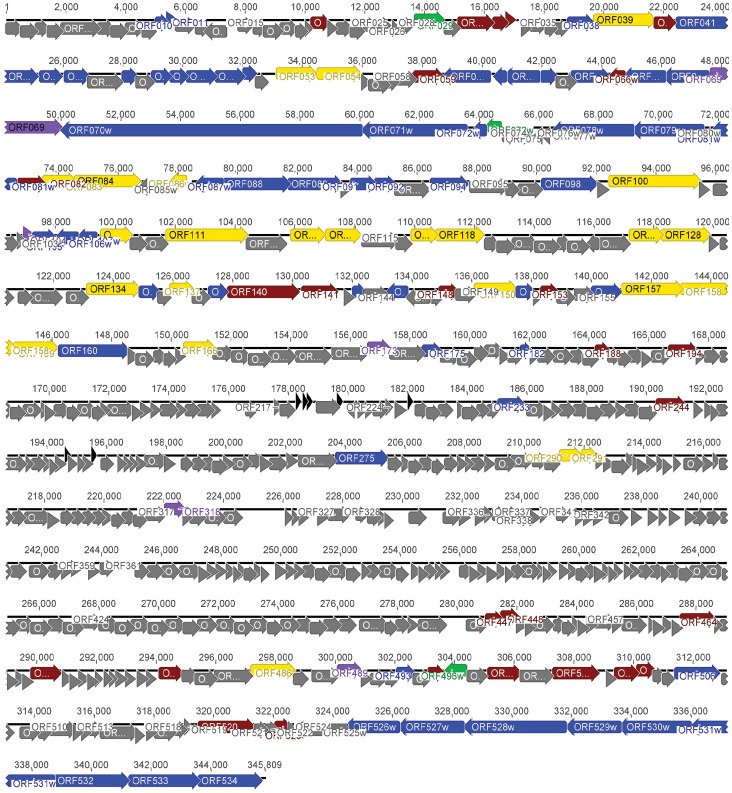
Functional genome map of bacteriophage RaK2. The coding capacity of the RaK genome is shown. Functions are assigned according to the characterized ORFs in NCBI database and/or MS/MS analysis. The colour code is as follows: yellow – DNA replication, recombination, repair and packaging; brown – transcription, translation, nucleotide metabolism; blue – structural proteins; purple – chaperones/assembly; green – lysis, host or phage interactions; grey – ORFs of unknown function; black – tRNA and pseudo-tRNA.

Phage genomes are usually organized into modular structures, with each module containing clusters of genes with specific functions [51], [52], [53]. In the case of RaK2, the precise delineation of the groups of genes is not possible because of the presence of many ORFs with no homology or those that encode proteins of unknown function. However, all structural proteins detected in the virus particle and/or identified by bioinformatics approach were mapped to at least two genome clusters: a large, ∼82 kb cluster encoding most of the structural components of the virion and a small, ∼20 kb cluster represented by tail fiber/tail spike genes (Fig. 4).

**Figure 5:**
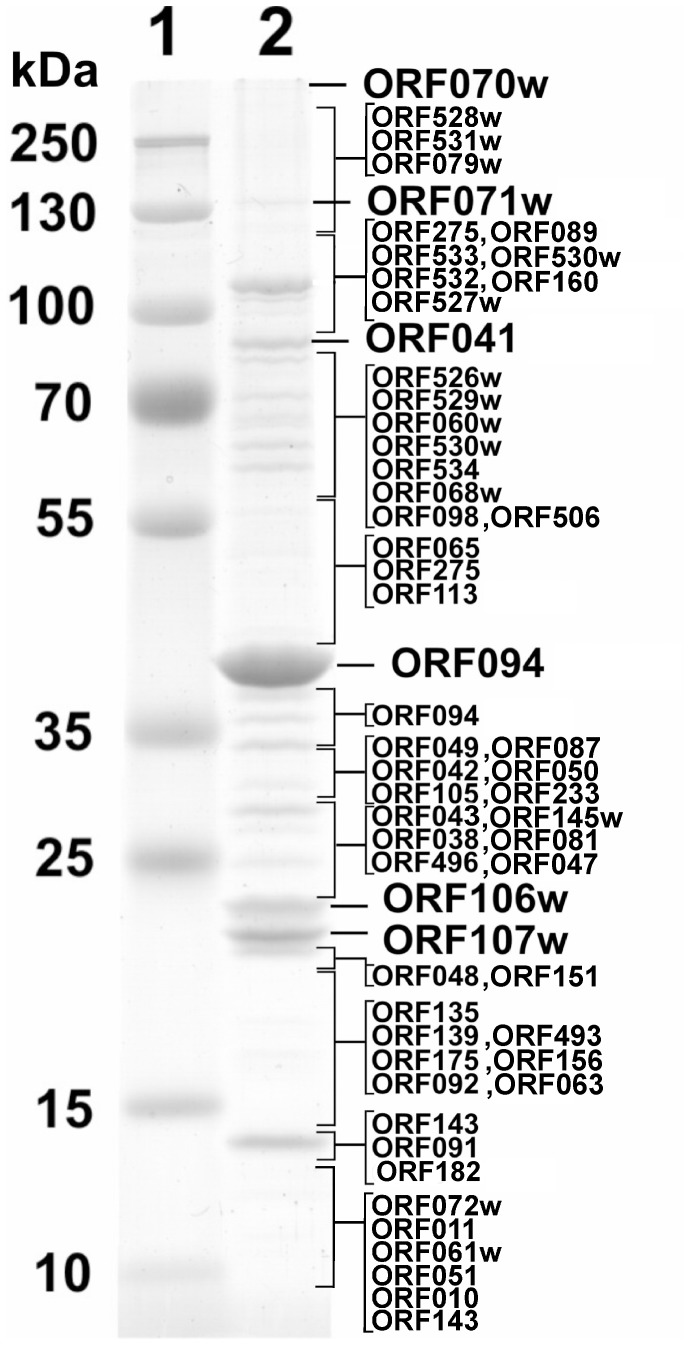
SDS-PAGE of virion proteins of RaK2. Lanes: 1– molecular mass marker Page Ruler™ prestained Protein Ladder Plus (Thermofisher), 2– phage RaK2. Relative migrations of MW marker proteins are indicated on the left. Proteins identified by MS/MS are indicated on the right.

As it can be seen in Fig. 5, the most abundant protein in the Rak2 virion is the major capsid protein gp094, which was found to migrate with a molecular mass slightly lower than predicted (∼43 kDa). The BLASTP search within the NCBI database revealed that the deduced amino acid sequence of gp094 of Rak2 shares 33% amino acid sequence identity with the major capsid protein of *Synechococcus* phage S-CBM2, and has homology to several T4-related gp23-like proteins. The N-terminal part of the major capsid protein of T4 is cleaved off during maturation of the capsid [54]. Hence, the SDS-PAGE migration of gp23 of Rak2 to a lower molecular weight than predicted may be an indication that this protein undergoes proteolytic cleavage. This observation is supported by the identification of a phage-encoded protease – a product of Rak2 ORF092. In addition, traces of the major capsid protein have been identified throughout the lower part of the gel suggesting that aspecific retention and partial degradation of this abundant protein may also occur.

The contractile tail sheath protein gp041 as well as the proteins encoded by ORF106w, ORF107w and ORF528w are also detected in the virion of Rak2. The gene product encoded by ORF528 (1113 aa) shares two regions of sequence similarity (amino acids 107–231 and 182–292) with tail fibre protein (p38) of T7-like phage SP6. Meanwhile gp041 (888 aa, 97.19 kDa) of Rak2 shares only one region (amino acids 706–878) of low sequence identity with the tail sheath protein of T4-related *Prochlorococcus* phage P-SSM2. The contractile tail of phage T4 is made of a baseplate and a tail tube (composed of 144 subunits of 18.45 kDa protein, gp19), which is surrounded by a tail sheath (composed of 138 copies of 71.33 kDa protein, gp18) [55]. The tail sheath protein of Rak2 is ∼18 kDa larger than that of T4 suggesting that the tail tube protein of Rak2 is also larger than its T4 counterpart, as it was observed in other myoviruses [56]. Unfortunately, bioinformatics analysis did not allow the identification of the tail tube protein of Rak2. However, the products of ORF106w (24,6 kDa) and ORF107w (21.1 kDa), two distantly related proteins that have no homologues in other phages except hypothetical proteins encoded by ORF264 and ORF265 of vB_CsaM_GAP32 respectively, show high propensity to form β-sheets. Hence, based on the results of the protein secondary structure prediction, the size and the abundance of gp106w/gp107w, we suggest that one of these gene products may function as the tail tube protein of Rak2. It is worth noting that in the case of all proteins mentioned above, there was a close match between the position of the protein on the gel and the predicted molecular mass of the protein suggesting that these peptides are not subjected to posttranslational-cleavage during Rak2 virion maturation.

Despite the high number of virion proteins recovered, four structural proteins of Rak2 predicted by genomic analysis (gp078, gp067, gp062 and gp088) were not detectable by mass spectrometry.This may be due to the incompatibility of these proteins with sample preparation procedures or/and because of their low abundance in virions (e.g. only six copies of the tail sheath completion protein (gp15) and three copies of baseplate-associated lysozyme (gp5) are present within the mature T4 virion [55]. In addition, functional assignment of gp062 and gp088 has been made on the basis of detectable, yet very low sequence similarity to biologically defined proteins suggesting that these gene products may not be associated with Rak2 morphogenesis at all.

## Discussion

In this paper we present the analysis of *Klebsiella* sp. infecting bacteriophage Rak2. With a genome size larger than that of most myoviruses, Rak2 is a perfect example of a “singleton” phage and thus presents a new genome type not closely related at the nucleotide level to any previously sequenced bacteriophages. In total, 272 out of 534 predicted RaK2 ORFs are “orphans” and 145 ORFs code for hypothetical proteins with no identifiable function assigned. Interestingly, the remaining 117 functionally annotated RaK2 genes show detectable amino acid sequence homology to proteins in a broad phylogenetic range of microorganisms, including Myovirus, Podovirus, and Siphovirus gene products, bacterial proteins as well as a dNMP kinase from eukaryotic *Cafeteria roenbergensis* virus BV-PW1. Similar to what has been observed in other jumbo bacteriophages [6], nearly 10% of Rak2 gene products are paralogues of other proteins of Rak2. Many of these paralogues are proteins that either have no reliable identity to database entries or are much more closely related to different Rak2 proteins than to those from other sequenced genomes.

**Figure 6:**
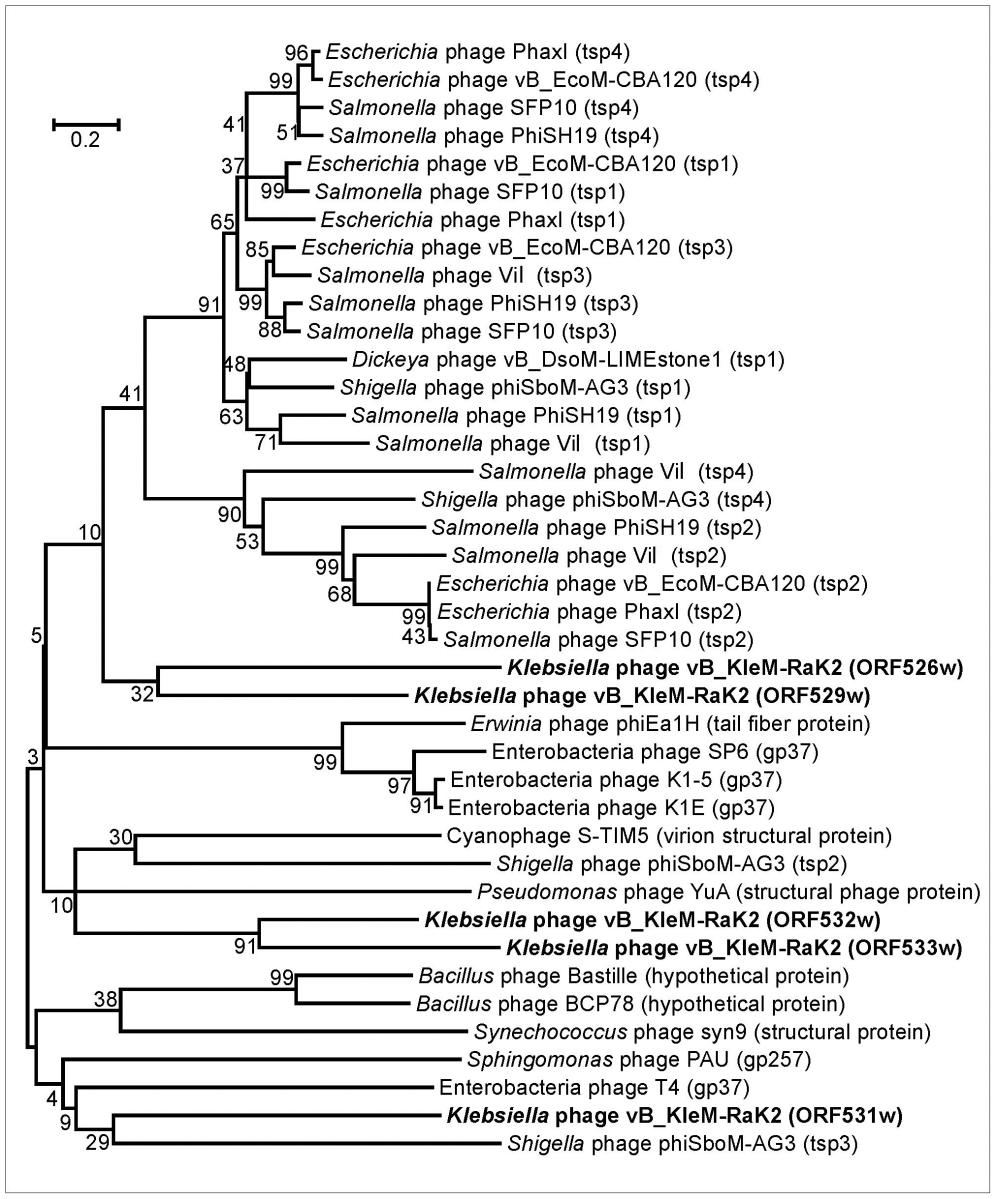
Neighbor-joining tree based on the alignment of the tail spike/tail fiber proteins from various phage genomes. The bootstrap values indicated.

Phage Rak2 is an atypical myovirus with six spiked tail fibers (Fig. 1.E) that appear to be formed predominantly from podovirus-like proteins. The electron micrographs suggest, prima facie, that the tail structure of Rak2 is similar to that from viunalikeviruses. Upon closer inspection, however, it is obvious that unlike viunalikeviruses, Rak2 does not possess folded prong-like structures. Moreover, the number of filaments (spikes) decorating tail fibers of Rak2 (as well as the complicated structure that they make) clearly differs from that of viunalikeviruses. According to E. M. Adriaenssens and co-authors [38], most of the identified ViI-like phages encode four tail spike proteins (TSP1-4). While TSP1-3 have a number of salient shared features and modules, the N-terminal region of TSP4 encodes a novel base-plate-binding protein found only in the ViI-like phages. Bacteriophage Rak2 harbours at least three potential tail spike genes (ORF531, ORF532 and ORF533) that all share significant amino acid sequence homology (Table S2). Based on the results of MS/MS analysis, all proteins mentioned are present in Rak2 virion, yet none of them shares the highest percentage amino acid sequence identity with the tail spike proteins of viunalikeviruses. Moreover, the phylogenetic analysis of the tail spike/tail fiber proteins from various phage genomes (Fig. 6), including podoviruses, viunalikeviruses as well as gp531, gp532 and gp533 of RaK2 suggests divergent evolution of these proteins.

Nevertheless, the MEME analysis revealed that two other structural proteins of Rak2, namely gp526 and gp529, share a conserved N-terminal motif (M[IN][SP]QF[NAS]QP[KR]GS[TV]S[IK]E[VT]NK[QD]SIAR[NK]FG[VC]K[EK][DS]EV[VIL]Y[FA][KST][AS]G[IQ][DS]L[ST]G[YF]KVIYD[EK]) with TSP1 and TSP3 of viunalikeviruses. Interestingly, TSP1 and TSP3 share a common N-terminal region that may represent the domain for binding to the phage base-plate [38]. Hence, although the sequence similarity shared by gp526, gp529 and TSPs is limited to a region of about 50 N-terminal amino acids, such evolutionary conservation suggests its functional importance.

Our results suggest that RaK2 likely encodes 10 mostly podovirus-related tail spike/tail fiber proteins (gp098, gp526-gp534) that, according to MS/MS analysis, all are present in mature virus particles. Nevertheless, i) which protein is responsible for the recognition and binding of RaK2 to the host cell, or ii) exactly how many proposed tail proteins are directly involved in the aforementioned processes – these are the questions we are unable to answer at the moment.

As it was mentioned previously, the major head protein of Rak2 as well as a number of other morphogenesis-related gene products have homology to those from several T4-related bacteriophages. In addition, phage Rak2 encodes a number of distant homologues of T4-like proteins associated with DNA replication, recombination, repair and nucleotide metabolism. All of these proteins are encoded by genes that belong to the “core genome” shared within subsets of T4-related phages. Relatives of *Escherichia coli* phage T4 constitute one of the most ecologically and genomically diverse groups of phages known and include viruses infecting enterobacteria, *Acinetobacter*, *Aeromonas*, *Delftia*, *Vibrio*, *Prochlorococcus* and *Synechococcus* [57]. They all share a set of 38 conserved genes which constitute the T4 superfamily core genome [40]. In total, 15 of the 38 T4-related “core genes” (*3*, *8*, *14*, *16*, *19*, *22*, *25*, *33*, *34*, *35*, *36*, *53*, *62* and *reg*A) are missing from Rak2 suggesting that this phage is only distantly related to T4-like viruses.

**Figure 7:**
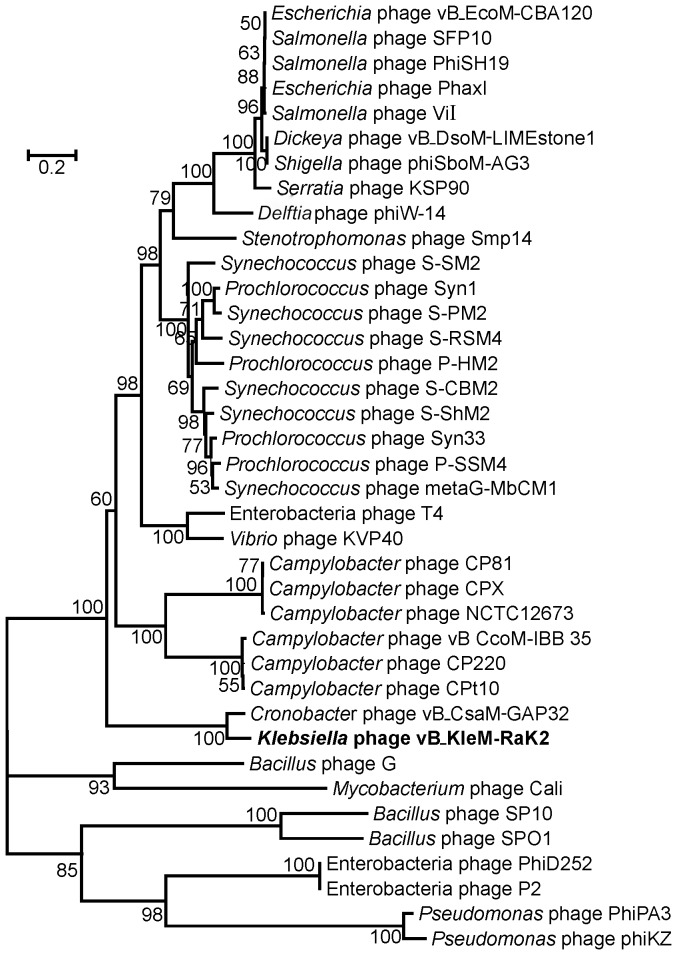
Neighbor-joining tree analysis based on the alignment of the amino acid sequences of the major capsid proteins from various myoviruses. The numbers at the nodes indicate the bootstrap probabilities.

**Figure 8:**
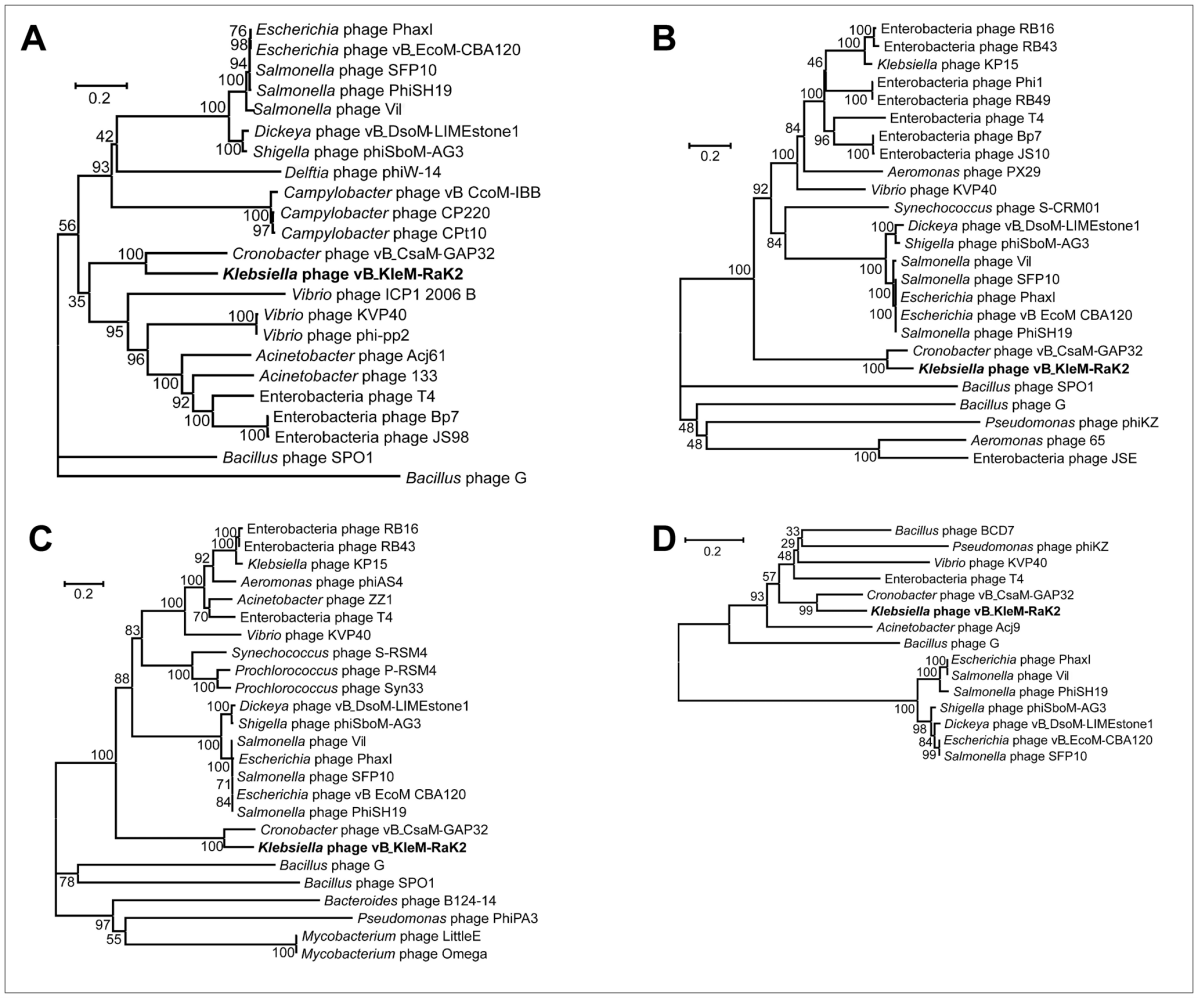
Phylogenetic analysis. Relationships of (A) DNA ligase; (B) DNA polymerase; (C) terminase large subunit; (D) thymidylate synthase across diverse phage types. The bootstrap values are indicated.

The bioinformatics analysis revealed no significant DNA sequence conservation between Rak2 and other myoviruses, except phage vB_CsaM_GAP32. Hence, the phylogenetic analysis of five conserved proteins – the major capsid protein (Fig. 7), DNA ligase, DNA polymerase, terminase large subunit and thymidylate synthase (Fig. 8) – has been carried out to better understand the evolutionary relationships between these phages. All five phylogenetic trees show that Rak2 represents an evolutionarily distinct branch of the *Myoviridae*. Moreover, the high divergence of all Rak2 genes indicates that this virus is a representative of an anciently branched group of phages that have been relatively isolated from horizontal exchange with other known phage groups.

As it was mentioned above, the genomes of RaK2 and vB_CsaM_GAP32 share several regions of DNA sequence conservation, accounting for approximately 28% of RaK2 DNA. In addition, both genomes share a set of 213 genes (∼39% of RaK2 total coding capacity), including 104 RaK2 ORFs that have been functionaly annotated (amino acid identity ranging between 78% for aerobic ribonucleoside diphosphate reductase subunit beta (RaK2 gp141) and 29% for RaK2 gp051, virion structural protein identified by MS/MS). In total, 11 out of 16 functionally assigned genes in RaK2, which have no vB_CsaM_GAP32 homologues, are those coding for virion structural proteins. In fact, nearly all homologues of RaK2 tail fiber/tail spike proteins are missing from vB_CsaM_GAP32 suggesting that in the case of these two phages not only the architecture of virion tails may be different, but the host cell attachment/recognition processes as well. As may be seen in Fig. 7 and Fig. 8, bacteriophages RaK2 and vB_CsaM_GAP32, although possibly related, represent distinct evolutionary branches. However, at present, the exact phylogenetic relationships between RaK2 and vB_CsaM_GAP32 are ambigous, because no data regarding the morphology or physiology of the latter phage are available yet.

### Conclusions

We conclude that the genome (345,809 bp encoding 534 ORFs) and morphological characteristics of bacteriophage RaK2 are vastly different from previously studied dsDNA phages. In addition, our results suggest that phage RaK2 represents an evolutionarily distinct branch of the *Myoviridae*.

## Supporting Information

Table S1 **Bacterial strains used in this study.**
(PDF)

Table S2 **The list of RaK2 ORFs with functional assignment.**
(PDF)

Table S3 **RaK2 virion proteins identified by MS/MS.**
(PDF)
