# Correction: *Klebsiella* Phage vB_KleM-RaK2 — A Giant Singleton Virus of the Family *Myoviridae*

**DOI:** 10.1371/annotation/a1d15675-2942-41ba-92f4-3dad6bc6cac6

**Published:** 2013-12-17

**Authors:** Eugenijus Šimoliūnas, Laura Kaliniene, Lidija Truncaitė, Aurelija Zajančkauskaitė, Juozas Staniulis, Algirdas Kaupinis, Marija Ger, Mindaugas Valius, Rolandas Meškys

The name of a funding organization is given incorrectly in the Funding Statement. The Funding Statement should read: "This work was funded by the Research Council of Lithuania (SVE-04/2012). URL: http://www.lmt.lt/."

