# Supplementary material for: Klebsiella Phage vB_KleM-RaK2 — A Giant Singleton Virus of the Family Myoviridae
Source: PLoS One. 2013 Apr 9;8(4):e60717. doi: 10.1371/journal.pone.0060717 (PMC3622015; doi:10.1371/journal.pone.0060717)
Supplement: Table S1 — Bacterial strains used in this study. (PDF) [file pone.0060717.s001.pdf]

TABLE S1. Bacterial strains used in this study.

Strain	Relevant characteristics	Source or reference
Acinetobacter baumannii #16, #46		Prof. E. Suziedeliene
Acinetobacter sp. 3#9, 13#23		Prof. E. Suziedeliene
Arthrobacter globiformis NRRL B-2979	wild type	Casaite et al. 2011
Arthrobacter crystallopoietes DSM 20117	type strain	DSMZ
Arthrobacter sp. BL-3, PY-21, PY-22	environmental isolates	Stanislauskiene et al. 2012
Buttiauxella sp. S1-1	environmental isolate	This study
Citrobacter freundii		Prof. E. Suziedeliene
Enterobacter sp. VT1-1	environmental isolate	This study
Enterobacter cloacae		Prof. E. Suziedeliene
Erwinia carotovora 8982, 961-63		Prof. E. Suziedeliene
Escherichia coli B^E	sup^0	Dr. L. W. Black
Escherichia coli BL21 (DE3)	F^- dcm ompT hsdS(rB^-mB^-) gal λ(DE3)	Avidis
Escherichia coli B834 (DE)	F^- hsdS metE gal ompT	Dr. L. W. Black
Escherichia coli CR63	supD, ser	Dr. K. N. Kreuzer
Escherichia coli DH5α	F^- endA1 glnV44 thi-1 recA1 relA1 gyrA96 deoR nupG Φ80dlacZΔM15 Δ(lacZYA-argF)U169, hsdR17(r_K^- m_K^+), λ–	Pharmacia

Escherichia coli DH10β	F^- endA1 recA1 galE15 galK16 nupG rpsL ΔlacX74 Φ80lacZΔM15 araD139 Δ(ara,leu)7697 mcrA Δ(mrr-hsdRMS-mcrBC) λ^-	Invitrogen
Escherichia coli GM2163	dam-13::Tn 9 dcm-6 hsdR2 leuB6 his-4 thi-1 ara-14 lacY1 galK2 galT22 xyl-5 mtl-1 rpsL136 tonA31 tsx-78 supE44 $McrA^-$ $McrB^-$	Fermentas
Escherichia coli JM109	endA1 glnV44 thi-1 relA1 gyrA96 recA1 $mcrB^+$ Δ(lac-proAB) e14- [F' traD36 $proAB^+$ $lacI^q$ lacZΔM15] hsdR17($r_K^- m_K^+$)	Fermentas
Escherichia coli MH1	araD139 ΔlacX74 $galU^-$ $galK^-$ hsr hsm rpsL	Dr. K. N. Kreuzer
Escherichia coli Nova Blue (DE3)	endA1 hsdR17($r_{K12}^- m_{K12}^+$) supE44 thi-1 recA1 gyrA96 relA1lac [$F^/$ $proA^+B^+$ $lacI^q$ZΔM15::Tn10 (Tet^r)]	Avidis
Escherichia coli XL1 Blue	endA1 gyrA96(nal^r) thi-1 recA1 relA1 lac glnV44 F'[::Tn10 $proAB^+$ $lacI^q$ (lacZ)M15 Amy Cm^r]	Stratagene

	hsdR17(r_K^- m_K^+) (Tet[r])	
Klebsiella sp. KV-1	environmental isolate	This study
Klebsiella sp. KV-3	phage host, pig farm sewage, Amp[r], Str[r], Tet[r], Kan[s], Gm[s], Nc[s],Cl[r/s]	This study
Klebsiella pneumoniae ATCC BAA – 1705		ATCC
Klebsiella oxytoca ATCC 8724		ATCC
Klebsiella pneumoniae 279		Prof. E. Suziedeliene
Pseudomonas aeruginosa PAO1		Prof. E. Suziedeliene
Pseudomonas brenneri D14		Prof. E. Suziedeliene
Pseudomonas sp. PV1-1, RA1-1, RA1-3, RA1-11	environmental isolates	This study
Rhodococcus erythropolis SQ1		Quan and Dabbs, 1993
Rhodococcus sp. PY11	environmental isolate	Semėnaitė et al. 2000
Salmonella enterica ser. *Typhimurium* 292		Prof. E. Suziedeliene

Strains designated “this study” were isolated in Lithuania. To identify bacterial isolates 16S rRNA gene-based sequence analysis was performed. Amp – ampicillin; Kan – kanamycin; Str – streptomycin; Tc – tetracycline; Gm – gentamicin; Nc – neomycin; Cm – chloramphenicol; Nal – nalidixic acid; [r] – resistant; [s] – susceptible.
