# Supplementary material for: Klebsiella Phage vB_KleM-RaK2 — A Giant Singleton Virus of the Family Myoviridae
Source: PLoS One. 2013 Apr 9;8(4):e60717. doi: 10.1371/journal.pone.0060717 (PMC3622015; doi:10.1371/journal.pone.0060717)
Supplement: Table S2 — The list of RaK2 ORFs with functional assignment. (PDF) [file pone.0060717.s002.pdf]

TABLE S2. The list of RaK2 ORFs with functional assignment.

RaK2 ORF (protein length aa)	Predicted product	Reference organism locus taq (protein length aa)	Amino acid sequence identity /similarity (length of the overlapping segment)	*E-value*	Homologue in *Cronobacter* phage vB_CsaM_GAP32
ORF010 (123)	probable phage protein	*Neisseria meningitidis* ATCC 13091 HMPREF0602_0060 (106)	32/55 (112)	7e-10	AFC21610.1
ORF011 (105)	hypothetical protein	*Erwinia* phage phiEa21-4 g13 (91)	32/53 (96)	0.96	AFC21611.1
ORF021 (192)	putative thymidine kinase	Enterobacteria phage phi92 PHI92_080 (187)	49/69 (189)	7e-53	AFC21620.1
ORF029 (300)	phage lysozyme family protein	*Shigella sonnei* 53G SS53G_3452 (149)	37/51 (158)	9e-19	AFC21630.1
ORF031 (368)	multifunctional CCA protein	*Coxiella burnetii* 'MSU Goat Q177' (376)	46/62 (274)	5e-68	AFC21636.1
ORF032 (174)	NudE nudix hydrolase	*Klebsiella* phage KP15 nudE (131)	42/61 (132)	8e-29	AFC21637.1
ORF033 (83)	glutaredoxin	*Pseudomonas* phage OBP OBP_012 (88)	49/66 (74)	3e-15	AFC21638.1
ORF038 (266)	neck protein	*Stenotrophomonas* phage Smp14 g13 (253)	27/56 (210)	8e-05	AFC21645.1

ORF039 (668)	possible homing endonuclease	*Campylobacter* phage CPt10 CPt10_0521 (574)	34/50 (596)	8e-61	-----
ORF040 (159)	putative deoxynucleoside monophosphate kinase	*Cafeteria roenbergensis* virus BV-PW1 crov080 (187)	41/64 (179)	1e-34	AFC21647.1
ORF041 (889)	phage tail sheath protein	*Syntrophobacter fumaroxidans* MPOB Sfum_3815(509)	35/49 (275)	4e-33	AFC21648.1
ORF042 (227)	----	----	----	----	AFC21649.1
ORF043 (257)	----	----	----	----	AFC21650.1
ORF045 (149)	head completion protein	*Synechococcus* phage S-SSM7 SSSM7_008 (159)	46/58 (140)	4e-29	AFC21652.1
ORF047 (206)	----	----	----	----	AFC21654.1
ORF048 (181)	----	----	----	----	AFC21655.1
ORF049 (315)	----	----	----	----	AFC21656.1
ORF050 (304)	----	----	----	----	AFC21657.1
ORF051 (134)	----	----	----	----	AFC21658.1
ORF053 (445)	ATPase	*Sulfuricurvum kujiense* DSM 16994 Sulku_0344 (330)	31/47 (404)	2e-43	AFC21660.1

ORF054 (462)	metallopeptidase-related protein	Desulfovibrio alaskensis G20 Dde_0484 (444)	28/41 (482)	1e-44	AFC21661.1
ORF059 (310)	thymidylate synthase	*Halorhodospira halophila* SL1 Hhal_2047 (264)	48/60 (309)	3e-93	AFC21667.1
ORF060w (510)	----	----	----	----	AFC21668.1
ORF061w (115)	----	----	----	----	AFC21671.1
ORF062w (371)	Virion structural protein	*Pseudomonas* phage 201phi2-1 201phi2-1p230 (1480)	24/53 (255)	0.014	AFC21672.1
ORF063 (165)	----	----	----	----	AFC21673.1
ORF065 (410)	----	----	----	----	AFC21675.1
ORF066w (157)	MutT/NUDIX family proteint	*Ralstonia* phage RSL1 RSL1_ORF109 (167)	36/50 (121)	6e-12	AFC21676.1
ORF067w (441)	tail sheath stabilizer and completion protein	Enterobacteria phage RB49 RB49p151 (277)	28/46 (159)	0.001	AFC21677.1
ORF068w (486)	----	----	----	----	AFC21678.1
ORF069 (769)	ClpA-like protein	*Gluconacetobacter* sp. SXCC-1 SXCC_03678(775)	40/61 (757)	0.0	AFC21679.1
ORF070w (3326)	-----	-----	-----	-----	AFC21680.1

ORF071w (1154)	baseplate wedge	*Synechococcus* phage S-SM1 Ppro_1814 (814)	33/56 (95)	4e-04	AFC21681.1
ORF072w (129)	phage baseplate assembly protein	*Natrinema sp. J7-2* NJ7G_0012 (132)	35/53 (86)	2e-04	AFC21682.1
ORF073w (162)	phage lysozyme	*Salmonella enterica subsp. enterica* ZP_03216458.1| (150)	47/65 (145)	5e-36	AFC21683.1
ORF078w (886)	baseplate hub subunit and tail lysozyme	*Klebsiella* phage KP15 gp157 (589)	28/44 (237)	1e-10	AFC21684.1
ORF079w (777)	-----	-----	-----	-----	AFC21685.1
ORF081w (243)	-----	-----	-----	-----	AFC21687.1
ORF082 (292)	sigma factor for late transcription	*Prochlorococcus* phage P-SSM4 PSSM4_118 (158)	29/55 (99)	7e-07	AFC21688.1
ORF083 (346)	recombination endonuclease subunit	*Synechococcus* phage S-SSM7 SSSM7_157(347)	33/50 (232)	9e-18	AFC21689.1
ORF084 (706)	endonuclease	*Synechococcus* phage S-CRM01 SCRM01_055 (574)	34/51 (250)	1e-22	AFC21690.1
ORF086 (164)	endoVII packaging and recombination endonuclease	*Salmonella* phage PVP-SE1 ORF126 (197)	39/55 (131)	1e-18	AFC21691.1
ORF087w (286)	Baseplate hub subunit	Synechococcus phage S-PM2 g26 (238)	24/57 (144)	4.7	AFC21693.1

ORF088 (721)	Phage protein, putative minor tail protein	*Weissella thailandensis fsh4-2* WT2_00250 (1150)	21/55 (597)	0.79	AFC21694.1
ORF089 (577)	portal vertex protein	*Vibrio* phage KVP40 KVP40.0358 (511)	36/53 (188)	5e-27	AFC21695.1
ORF091 (257)	----	----	----	----	AFC21697.1
ORF092 (206)	prohead core scaffold and protease	*Synechococcus* phage S-PM2 S-PM2p109 (214)	37/59 (164)	2e-25	AFC21698.1
ORF094 (393)	major capsid protein	*Synechococcus* phage S-CBM2 SWPG_00035 (545)	33/48 (401)	2e-44	AFC21700.1
ORF098 (595)	Putative tail fiber protein	*Salmonella enterica* subsp. enterica (600)	23/55 (539)	2e-05	AFC21704.1
ORF100 (1000)	DNA polymerase	*Vibrio* phage KVP40 KVP40.0082 (850)	26/42 (915)	7e-60	AFC21707.1
ORF104 (99)	10 kDa chaperonin GroS	*Coxiella burnetii* 'MSU Goat Q177' A35_A1822 (96)	42/63 (97)	3e-12	AFC21712.1
ORF105 (235)	----	----	----	----	AFC21713.1
ORF106w (1039)	----	----	----	----	AFC21714.1
ORF107w (202)	----	----	----	----	AFC21715.1
ORF108 (328)	RNaseH ribonuclease	*Aeromonas* phage Aes508 Aes508_202 (304)	28/46 (180)	3e-06	AFC21716.1

ORF111 (889)	terminase large subunit	*Ralstonia solanacearum* K60-1 RSK60_2210018 (1069)	28/46 (478)	1e-46	AFC21719.1
ORF113 (361)	single-stranded DNA binding protein	*Aeromonas* phage phiAS5 ORF106 (301)	24/52 (308)	0.7	AFC21721.1
ORF114 (370)	RecA-like recombination protein	*Synechococcus* phage syn9 BSV9_gp174 (336)	31/52 (302)	6e-35	AFC21722.1
ORF117 (273)	DNA polymerase III subunit epsilon-like protein	*Alicyclobacillus acidocaldarius subsp. acidocaldarius* DSM 446 Aaci_1500 (240)	27/44 (237)	4e-20	AFC21725.1
ORF118 (511)	RNA-DNA + DNA-DNA helicase	*Synechococcus* phage S-SM2 SSM2_124 (493)	30/49 (508)	3e-55	AFC21726.1
ORF127 (352)	DNA primase	*Methylobacterium* sp. GXF4 WYO_0834 (360)	29/45 (329)	5e-34	AFC21735.1
ORF128 (522)	ATP-dependent primase-helicase	*Campylobacter* phage vB_CcoM-IBB_35 gp41 (445)	27/47 (433)	8e-30	AFC21736.1
ORF134 (577)	Hef-like homing endonuclease	*Campylobacter* phage NCTC12673 NCTC12673_gp084 (484)	43/61 (319)	6e-61	----
ORF135 (207)	----	----	----	----	AFC21743.1

ORF137 (235)	Putative exonuclease	SAR86 cluster bacterium J5KER4_9GAMM (227)	39/61 (220)	7e-42	AFC21745.1
ORF139 (191)	----	----	----	----	AFC21747.1
ORF140 (771)	aerobic ribonucleoside diphosphate reductase subunit alpha	*Oceanimonas* sp. GK1 GU3_11305 *(758)*	56/74 (740)	0.0	AFC21748.1
ORF141 (374)	nrdB gene product	*Alteromonas macleodii* str. 'Deep ecotype' MADE_1010775 (376)	54/77 (375)	3e-148	AFC21750.1
ORF143 (246)	----	----	----	----	AFC21753.1
ORF144 (246)	Phosphoribosyl-ATP pyrophosphohydrolase-domain containing protein	*Acanthamoeba polyphaga* lentillevirus (251)	30/47 (220)	7e-14	----
ORF145w (214)	----	----	----	----	AFC21755.1
ORF148 (190)	Dihydrofolate reductase type I	*Colwellia* phage 9A COPG_00031 (170)	40/58 (139)	2e-21	AFC21758.1
ORF150 (452)	ATP-dependent DNA helicase dda	*Sulfurimonas gotlandica* GD1 CBGD1_1394 (500)	31/62 (433)	1e-22	AFC21760.1
ORF151 (186)	----	----	----	----	AFC21761.1

ORF153 (169)	translation initiation factor IF-3	*Megasphaera micronuciformis* F0359 infC (167)	35/58 (163)	2e-17	AFC21763.1
ORF156 (305)	hypothetical protein	*Pseudomonas* phage 201phi2-1 201phi2-1p383 (87)	46/63 (63)	2e-06	AFC21767.1
ORF157 (656)	topoisomerase II large subunit	*Vibrio* phage KVP40 KVP40.0045 (597)	30/46 (656)	4e-52	AFC21769.1
ORF158 (569)	Hef-like homing endonuclease	*Campylobacter* phage CP220 CPT_0054c (574)	32/50 (478)	1e-48	----
ORF159 (468)	topoisomerase II small subunit	*Vibrio* phage KVP40 KVP40.0282 (428)	31/53 (338)	3e-39	AFC21770.1
ORF160 (775)	----	----	----	----	AFC21771.1
ORF166 (319)	gp44-like sliding clamp loader subunit	*Campylobacter* phage NCTC12673 NCTC12673_gp003 (319)	34/53 (316)	6e-35	AFC21777.1
ORF173 (227)	putative metallopeptidase	Bacteriophage T5 T5.087 (233)	30/49 (174)	2e-06	AFC21787.1
ORF175 (184)	hypothetical protein	*Vibrio* phage pVp-1 pVp-1_0108 (186)	40/56 (176)	1e-31	AFC21789.1
ORF182 (111)	----	----	----	----	----
ORF188 (137)	peptidyl-tRNA hydrolase	*Methanocaldococcus vulcanius* M7 Metvu_1090 (115)	34/49 (122)	3e-12	AFC21834.1

ORF194 (297)	putative DNA-methyltransferase	*Vibrio* phage ICP1_2006_D TUST1-191_01135 (332)	43/55 (337)	8e-71	AFC21842.1
ORF233 (278)	----	----	----	----	AFC21931.1|
ORF244 (298)	putative RNA polymerase sigma-70 subunit	*Verrucosispora maris* AB-18-032 VAB18032_29206 (333)	31/47 (269)	2e-19	AFC21949.1
ORF275 (565)	----	----	----	----	AFC21480.1
ORF290 (226)	endonuclease dda	*Dickeya dadantii* 3937 Dda3937_00087 (246)	42/62 (227)	6e-55	----
ORF291 (147)	DenV endonuclease	Bacteriophage vB_EcoM-VR7 VR7_gp129 (138)	65/80 (131)	3e-57	AFC21957.1
ORF318 (210)	putative peptidase	*Yersinia phage* phiR1-37 phiR1-37_gp097 (219)	44/62 (206)	2e-42	----
ORF447 (172)	putative N-6-adenine-methyltransferase	*Staphylococcus epidermidis* NIHLM057 HMPREF9989_11133 (142)	47/67 (141)	1e-34	AFC21516.1
ORF448 (178)	cytidyltransferase	*Prochlorococcus* phage P-SSM4 PSSM4_155 (383)	37/56 (183)	7e-25	AFC21517.1

ORF464 (381)	RNA ligase 1 and tail fiber attachment catalyst	*Aeromonas* phage 44RR2.8t 44RRORF204c (383)	38/57 (361)	7e-67	AFC21528.1
ORF470 (314)	polynucleotide 5'-kinase and 3'-phosphatase PseT	*Aeromonas* phage Aeh1 Aeh1p282 (305)	35/52 (324)	7e-44	AFC21532.1
ORF482 (248)	putative serine/threonine protein phosphatase	*Escherichia* phage phAPEC8 phAPEC8_0086 (247)	38/58 (216)	6e-45	AFC21543.1
ORF486 (467)	ATP-dependent DNA ligase	*Vibrio* phage phi-pp2 pp2_055 (447)	31/54 (460)	6e-60	AFC21547.1
ORF489 (249)	ATP-dependent Clp protease proteolytic subunit	*Alkaliphilus oremlandii* Clos_2174 clpP(195)	46/62 (198)	4e-44	AFC21551.1
ORF493 (182)	----	----	----	----	----
ORF495 (156)	dCMP deaminase	*Acinetobacter* phage Acj9 Acj9p214 (181)	44/64 (162)	2e-38	AFC21557.1
ORF496 (212)	conserved structural protein contains bacterial Ig-like and invasin/intimin cell-adhesion domain	*Erwinia* phage phiEa116 g11600070 (206)	46/58 (130)	3e-17	AFC21559.1
ORF498 (354)	bifunctional nicotinamide mononucleotide adenylyltransferase/ AD P-ribose pyrophosphatase	*Burkholderia sp.* 383 Bcep18194_B0825 (346)	39/57 (349)	4e-68	AFC21560.1

ORF500 (490)	putative nicotinate phosphoribosyltransferase	*Psychrobacter arcticus* 273-4 Psyc_1914 (465)	36/53 (481)	3e-80	AFC21562.1
ORF502 (245)	NAD-dependent deacetylase	*Marinobacterium stanieri* S30 MstaS_010100017014 (249)	35/57 (193)	5e-28	AFC21568.1
ORF503 (127)	nudix hydrolase	*Aeromonas* phage PX29 PX29p330 (186)	33/49 (147)	3e-10	AFC21569.1
ORF506 (479)	PhoH-like protein	*Oceanospirillum* sp. MED92 MED92_02121 (464)	40/61 (490)	5e-113	AFC21571.1
ORF520 (601)	nrdD-like anaerobic NTP reductase	Enterobacteria phage phi92 PHI92_192A (599)	59/75 (602)	0.0	AFC21588.1
ORF523 (156)	anaerobic NTP reductase activating protein	*Clostridiales* bacterium 1_7_47_FAA CBFG_01379 (174)	51/71 (157)	1e-49	AFC21591.1
ORF526w (580)	hypothetical protein	*Pantoea sp.* At-9b Pat9b_0331 (534)	56/75 (476)	0.0	----
ORF527w (715)	gp17	*Klebsiella* phage K11 ASC_0042 (875)	24/43 (597)	1e-26	----
ORF528w (1114) (41% aa identity with RaK2 ORF533)	tail fiber fragment	Enterobacteria phage SP6 SP6p38 (320)	28/55 (132)	2e-05	----

ORF529w (584) (15% aa identity with RaK2 ORF526w)	hypothetical protein	*Klebsiella pneumoniae subsp. pneumoniae* WGLW5 HMPREF1308_05361	35/56 (501)	6e-88	----
ORF530w (779)	hypothetical protein (similar to tail fiber protein of phage vB_EcoP_G7C)	*Enterobacter* phage IME11 IME11_90 (896)	46/63 (542)	3e-153	----
ORF531w (895) (40% aa identity with RaK2 ORF532)	hypothetical protein (similar to tail fiber protein of phage K1-5)	*Burkholderia phytofirmans* PsJN Bphyt_6273 (503)	30/46 (445)	2e-30	----
ORF532 (807)	tailspike protein	Bacteriophage K1-5 EPKV1_gp40 (320)	30/50 (114)	1e-04	----
ORF533 (768) (47% aa identity with RaK2 ORF532)	hypothetical protein	*Klebsiella pneumoniae* 342 KPK_1664 (582)	34/50 (464)	5e-57	----
ORF534 (688) (33% aa identity with RaK2 ORF531)	hypothetical protein	*Vibrio* phage ICP2 ViPhICP2p14 (542)	33/56 (155)	9e-20	----

- functional assignment has been made based on the similarity to biologically defined proteins and/or MS/MS analysis
- podovirus-like proteins are shaded in grey, siphovirus-like in light blue.
