# Supplementary material for: Klebsiella Phage vB_KleM-RaK2 — A Giant Singleton Virus of the Family Myoviridae
Source: PLoS One. 2013 Apr 9;8(4):e60717. doi: 10.1371/journal.pone.0060717 (PMC3622015; doi:10.1371/journal.pone.0060717)
Supplement: Table S3 — RaK2 virion proteins identified by MS/MS. (PDF) [file pone.0060717.s003.pdf]

Table. S3 RaK2 virion proteins identified by MS/MS.

Gene product	Proposed function	MW (kDa)	Sequence coverage (%)	Number of unique peptides
gp010*	putative virion protein	13.932	18.0	3
gp011	putative virion protein	12.065	42.9	4
gp038*	neck protein	30.294	14.3	4
gp041*	tail sheath protein	97.191	28.3	21
gp042	putative virion protein	24.950	23.3	5
gp043	putative virion protein	28.720	17.1	3
gp047	putative virion protein	23.356	18.9	4
gp048	putative virion protein	20.374	18.8	3
gp049	putative virion protein	34.878	17.5	7
gp050	conserved phage protein	33.576	5.3	1
gp051	putative virion protein	14.727	18.7	2
gp060w	putative virion protein	56.225	12.5	5
gp061w	putative virion protein	12.577	26.1	2
gp063	putative virion protein	19.035	10.3	2
gp065	putative virion protein	45.317	13.7	5
gp068w	putative virion protein	56.144	8.4	4
gp070w	putative virion protein	382.000	2.2	7
gp071w*	baseplate wedge subunit	131.630	18.8	16
gp072w*	baseplate protein	15.024	23.3	3
gp079w	putative virion protein	87.857	13.4	9
gp081w	putative virion protein	27.440	6.6	2
gp087*w	baseplate protein	32.543	10.5	3
gp089*	portal vertex protein	64.744	13.5	7
gp091	putative virion protein	28.205	16.7	5
gp092	prohead core scaffolding protein and protease	22.870	26.7	6
gp094*	major capsid protein	42.756	26.5	10
gp098*	tail fiber protein	62.869	3.7	2

gp105	putative virion protein	27.854	21.7	5
gp106w	putative tail protein	24.651	55.4	13
gp107w	putative tail protein	21.133	53.5	11
gp113	putative single-stranded DNA binding protein	40.488	28.5	11
gp135	putative virion protein	23.259	36.2	7
gp139	putative virion protein	21.243	31.9	5
gp143	conserved phage protein	14.942	7.9	1
gp145w	putative virion protein	25.061	10.7	2
gp151	putative virion protein	20.561	25.8	4
gp156	putative virion protein	34.198	6.2	2
gp160	putative virion protein	87.718	8.1	6
gp175	putative virion protein	21.079	11.4	2
gp182	conserved phage protein	13.142	9.0	1
gp233	putative virion protein	30.971	13.7	4
gp275	putative virion protein	64.095	34.3	20
gp493	putative virion protein	21.475	17.0	2
gp496*	conserved virion protein	22.787	22.2	4
gp506	hypothetical protein	53.675	6.1	4
gp526w*	putative tail protein	63.021	28.8	15
gp527w*	tail fiber protein	79.449	14.3	7
gp528w*	tail fiber protein	121.755	12.4	12
gp529w*	putative tail protein	62.412	15.2	8
gp530w*	tail protein	86.033	6.3	5
gp531w*	putative tail protein	97.727	6.8	4
gp532*	tailspike protein	87.260	11.4	8
gp533*	putative tail protein	82.794	13.0	9
gp534*	putative virion protein	70.781	10.6	7

*– predicted to be virion proteins by bioinformatics approaches as well.
